# Anesthesia promotes acute expression of genes related to Alzheimer’s disease and latent tau aggregation in transgenic mouse models of tauopathy

**DOI:** 10.1186/s10020-022-00506-4

**Published:** 2022-07-20

**Authors:** John David Eun, Heidy Jimenez, Leslie Adrien, Adam Wolin, Philippe Marambaud, Peter Davies, Jeremy L. Koppel

**Affiliations:** 1 Donald and Barbara Zucker School of Medicine at Hofstra/Northwell, Hempstead, NY USA; 2 Litwin-Zucker Research Center for the Study of Alzheimer’s Disease, Feinstein Institutes for Medical Research, 350 Community Drive, 4th floor, Manhasset, NY 11030 USA; 3 Zucker Hillside Hospital, Donald and Barbara Zucker School of Medicine at Hofstra/Northwell, Great Neck, NY USA

**Keywords:** Alzheimer’s disease, Anesthesia, Isoflurane, Tau, Hyperphosphorylation, Neuroinflammation, Hibernation, Hypothermia

## Abstract

**Background:**

Exposure to anesthesia in the elderly might increase the risk of dementia. Although the mechanism underlying the association is uncertain, anesthesia has been shown to induce acute tau hyperphosphorylation in preclinical models. We sought to investigate the impact of anesthesia on gene expression and on acute and long-term changes in tau biochemistry in transgenic models of tauopathy in order to better understand how anesthesia influences the pathophysiology of dementia.

**Methods:**

We exposed mice with over-expressed human mutant tau (P301L and hyperdopaminergic COMTKO/P301L) to two hours of isoflurane and compared anesthetized mice to controls at several time points. We evaluated tau hyperphosphorylation with quantitative high-sensitivity enzyme-linked immunosorbent assay and performed differential expression and functional transcriptome analyses following bulk mRNA-sequencing.

**Results:**

Anesthesia induced acute hyperphosphorylation of tau at epitopes related to Alzheimer’s disease (AD) in both P301L-based models. Anesthesia was associated with differential expression of genes in the neurodegenerative pathways (e.g., AD-risk genes *ApoE* and *Trem2*) and thermogenesis pathway, which is related to both mammalian hibernation and tau phosphorylation. One and three months after anesthesia, hyperphosphorylated tau aggregates were increased in the anesthetized mice.

**Conclusions:**

Anesthesia may influence the expression of AD-risk genes and induce biochemical changes in tau that promote aggregation even after single exposure. Further preclinical and human studies are necessary to establish the relevance of our transcriptomic and biochemical findings in these preclinical models to the pathogenesis of dementia following anesthesia.

*Trial registration:* Not applicable.

**Supplementary Information:**

The online version contains supplementary material available at 10.1186/s10020-022-00506-4.

## Background

While causes of dementia are multifactorial, age is the strongest risk factor. The US prevalence of Alzheimer’s disease (AD), which accounts for 60–80 percent of dementia, was estimated to be 6.5 million in 2022, but its prevalence in different age groups ranges from 5.3% among age 65–74 to 34.6% among age over 85 (Rajan et al. 2021). In older populations, a modifiable risk factor might be exposure to general anesthesia and surgery. Exposure after age 60, but not in younger adults, has shown to be associated with greater acceleration of cognitive decline than expected for normal aging (Schulte et al. 2018; Sprung et al. 2016). Although an earlier meta-analysis of case–control studies found that exposure to general anesthesia and surgery did not increase the incidence of dementia (Seitz et al. 2011), two nationwide studies in Asia have since shown increased risk of dementia following such exposure (Chen et al. 2014; Kim et al. 2018).

Older adults might be at higher risk of post-operative cognitive decline because of pre-existing tau pathology, which increases with age (Lowe et al. 2018). The two pathological hallmarks of AD are extracellular plaques and intracellular neurofibrillary tangles (NFTs) that are composed of amyloid-β (Aβ) and tau, respectively. Unlike beta-amyloid pathology, the distribution and density of NFTs correlate with severity and duration of neurodegeneration and dementia (Arriagada et al. 1992; Gomez-Isla et al. 1997). Tau is a soluble, axonal protein that stabilizes microtubules and promotes their assembly (Drechsel et al. 1992). But in AD, tau undergoes aggregation via formation of paired helical filaments (PHFs) and then insoluble NFTs. Anesthesia and surgery have been shown to induce changes in biomarkers that reflect in vivo neuropathology. For instance, one study found that the post-operative level of CSF tau, not Aβ, was similar to the mean level in patients with AD (Berger et al. 2016). In another study, both plasma tau and a marker of axonal injury increased following anesthesia and surgery (Evered et al. 2018). However, the role of anesthesia alone in such acute response is unclear because surgery can activate peripheral stimuli that influence the central nervous system (Yang et al. 2020). No longitudinal study has examined whether these post-operative elevations of tau biomarkers lead to persistent development of pathological tau.

Animal models of tau pathology could be employed to investigate the impact of anesthesia on tau outcomes relevant to dementia and identify other molecular pathways affected by the anesthetic exposure. The tau in PHFs are conformationally altered and abnormally phosphorylated (“hyperphosphorylated”) (Goedert et al. 1988; Grundke-Iqbal et al. 1986). In contrast to wild-type mice, transgenic mouse models that express human tau develop tau aggregates (Lewis et al. 2000), and studies using antibodies that recognize phospho-epitopes in human NFTs have demonstrated that neurodegeneration follows the development of hyperphosphorylated tau pathology in the transgenic mice (Goedert et al. 2017). Both volatile and intravenous anesthetics—with or without temperature-control—have been found to induce tau hyperphosphorylation in both wild-type and transgenic mice (Planel et al. 2007, 2008, 2009; Tang et al. 2011a). Yet, the phospho-epitopes may be dephosphorylated as early as 2–6 h after anesthesia, and changes in activities of kinase and phosphatase that regulate tau phosphorylation are transient as well (Planel et al. 2007, 2009). Only two studies have sought to address the critical question of whether acute tau hyperphosphorylation is associated with downstream aggregation and had indeterminate findings (Planel et al. 2009; Tang et al. 2011a). Moreover, studies using transgenic models have not explored the acute effects of anesthesia on metabolic dysfunction and neuroinflammation, both of which are involved in AD pathogenesis. Anesthesia might disrupt mitochondrial function and allow infiltration of peripheral leukocytes and cytokines into the CNS (Maldonado 2018; Zhang et al. 2012), and the presence of nascent tau pathology may compound such effects (Bennett et al. 2018; Kopeikina et al. 2011).

In order to model the consequences of anesthesia in populations at risk for AD, we examined the short- and long-term effects of anesthesia on transgenic mouse models of tauopathy. We investigated broader cellular responses to anesthesia in the setting of acute tau hyperphosphorylation by using mRNA-sequencing and whether anesthesia-induced tau hyperphosphorylation translates into aggregation with high-sensitivity quantification of multiple phospho-epitopes. We compared the acute effects in JNPL3 (P301L) mice, which express the mutant human tau associated with autosomal dominant tauopathy (Lewis et al. 2000), to COMTKO/P301L mice, which have impaired dopamine metabolism (Koppel et al. 2019). Our group demonstrated that the COMTKO/P301L mice have dopamine-driven increases in tau phosphorylation relative to P301L mice (Jimenez et al. 2022). The degree of baseline tau hyperphosphorylation could be critical in determining the impact of anesthesia on the subsequent tau aggregation that may be relevant for post-operative cognitive decline. Also, cognitive decline often follows a course of delirium in the immediate aftermath of exposure to anesthesia (Inouye et al. 2016), and the risk is higher in those with dementia (Inouye et al. 2014). Increases in dopamine signaling has been directly associated with delirium for which dopamine-blocking antipsychotics are used as treatment (Burry et al. 2018; Ramírez-Bermúdez et al. 2019). Our selection of the COMTKO/P301L model is in anticipation of future preclinical paradigms that may elucidate molecular pathways in delirium and their relevance to dementia. We observed that anesthesia induces robust acute tau hyperphosphorylation, and as the hyperphosphorylation normalized 24 h after anesthesia, gene expression changes, including those of AD-related risk genes, persisted. Most importantly, following the mice up to three months after anesthesia revealed progressive increases in tau aggregation that could explain the chronic effects of anesthesia on patients with dementia.

## Materials and methods

### Animals

All animal protocols were approved by the Institutional Animal Care and Use Committee (IACUC) at the Feinstein Institutes for Medical Research and conformed to ethical standards. All mice were housed under normal conditions and had ad libitum access to food and water. JNPL3 (P301L) mice (research resource identifier, RRID:MGI3604148) express a mutant human tau (0N4R) under the mouse prion promoter at twice the level of endogenous murine tau (Lewis et al. 2000). P301L mice on a C57BL/6*DBA/2 background were obtained from Taconic Biosciences (Rensselear, NY). Catechol-*O*-methyltransferase (COMT) is involved in enzymatic degradation of dopamine. COMT-KO mice on a C57BL/6 background were previously crossed with P301L mice to produce a colony of COMT^−/−^ P301L^+/ +^ (COMTKO/P301L) mice in which tau phosphorylation increases in response to elevated dopamine (Koppel et al. 2019).

### Antibodies

All anti-tau monoclonal antibodies utilized in tau biochemistry were produced in the laboratory of Peter Davies. The following panel of antibodies detect epitopes that are relevant across a spectrum of AD severity. RZ3 (RRID:AB2716721) recognizes pThr231, found mainly in pre-tangles; CP13 (RRID:AB2314223) recognizes pSer202 and is used to detect early neuritic and advanced NFT pathology (Janocko et al. 2012); and PHF1 (RRID:AB2315150) recognizes pSer396/-404, found in diffuse and fibrillar tangles (Acker et al. 2013; Augustinack et al. 2002). Two pan-tau antibodies used were: DA9 (RRID:AB 2716723), which recognizes amino acids 102–150 and is conjugated with horseradish peroxidase [HRP), and DA31 (RRID:AB2716724), which recognizes amino acids 150–190 (Acker et al. 2013).

### Anesthesia

We selected specific age and sex of each mouse model so that we could distinguish the effects of anesthesia-induced tau hyperphosphorylation from natural history in long-term follow-ups. We anesthetized the mice at an age when neurofibrillary pathology begins to develop, which were 4–5 months for the mice with P301L tau (Lewis et al. 2000). We used only the male COMTKO/P301L and P301L mice because they develop tau pathology more slowly than their female counterparts (Buccarello et al. 2017). Male 5-month-old P301L mice and male 4-month-old COMTKO/P301L mice were exposed to 2 h of isoflurane (Henry Schein Animal Health, Dublin, OH) in oxygenated chambers without temperature-control (2.5% isoflurane for induction and 1.5% isoflurane during maintenance). Following the 2-h anesthesia, the mice were either immediately euthanized by cervical dislocation or returned to their cage to be euthanized at future time points. The mice in the control group were placed in the anesthesia chambers for five minutes, the approximate duration of induction, in order to control for any stress induced by the chambers. In all groups, the brain was removed and hemisected sagittally.

### Enzyme-linked immunosorbent assay (ELISA)

Following hemisection, the hippocampus and cortex were dissected from one hemisphere. Homogenized brain samples were stored at − 80 °C and processed as previously described (Acker et al. 2013). Heat-stable soluble tau preparations were added with 4% 5 M NaCl and 5% β-mercaptoethanol, boiled at 100 °C for 10 min, and cooled on ice for 15 min, and centrifuged at 14,000 rpm at 4 °C for 15 min. Supernatants were then collected. Insoluble tau preparations were centrifuged at 60,000 rpm at 4 °C for 10 min, and collected supernatants were centrifuged at 75,000 rpm at 4 °C for 30 min. Pellet was resuspended in the previously mentioned homogenizing buffer and centrifuged again at 75,000 rpm at 4 °C for 30 min. Pellet was resuspended in Laemmli sample buffer, a solution of Tris-buffered saline (TBS), pH6.8, containing 4% SDS, 2% β-mercaptoethanol, 10% glycerol, and bromophenol blue. Samples were boiled at 100 °C for 5 min before use in ELISA.

In contrast to Western blot or immunohistochemistry (IHC), ELISA is a quantitative method for measuring phospho-tau epitopes, and its superior sensitivity and lower tissue requirement allow for analysis of different regional dissections as well as both insoluble and tau fractions (Acker et al. 2013; Chai et al. 2011). The procedures for ELISA are described elsewhere (Koppel et al. 2019; Acker et al. 2013). Briefly, ninety-six-well plates (Fisher Scientific, Pittsburgh, PA) were coated with DA31, RZ3, CP13, and PHF1 at a concentration of 6 μg/mL in coating buffer for at least 48 h at 4 °C. After blocking and washing the plates, equal volumes of the sample (in duplicates) and DA9-HRP were added. The plates were read with an Infinite m200 plate reader (Tecan, Mannedorf, Switzerland) at 450 nm after overnight incubation at 4 °C on shaker. Phospho-tau/total tau ratios were calculated after normalization with protein concentrations of the brain homogenate lysates.

### RNA-sequencing

Total RNA was extracted from the forebrain with RNeasy Mini Kit (Qiagen, Hilden, Germany) according to the manufacturer’s protocol. NanoDrop ND-100 Spectrophotometer was used to determine the concentration of RNA samples (NanoDrop Technologies, Wilmington, DE), and BioAnalyzer RNA 2,100 kit was used to test their integrity (Agilent Technologies, Santa Clara, CA). RNA sequencing was performed with the Illumina mRNA TruSeq Stranded method on Illumina HiSeq (Illumina, San Diego, CA).

We used the RNA-seq Alignment app on Illumina BaseSpace Sequence Hub for the following functions: Bowtie for filtering the input reads; the STAR aligner for read mapping; Salmon for quantification of reference genes and transcripts; and Strelka Variant caller for variant calling. The UCSC mm10 *mus musculus* (with RefSeq gene annotation) was the reference genome. After downloading BAM output files, we performed the subsequent analyses in R. Counts of the aligned reads were generated using featureCounts in the Rsubread package (Liao et al. 2014). A pre-filter was applied so that only genes with at least ten counts total were analyzed. Differential expression analysis was performed using DESeq2 (Love et al. 2014). Log_2_-fold-change (LFC) estimates were shrunk using the *apeglm* method for improved ranking and visualization of genes (Zhu et al. 2018). *P*-values were adjusted for multiple-testing with the Benjamini–Hochberg procedure.

Normalized counts that were generated from DESeq2 were plotted with GraphPad Prism. We highlighted the following 11 murine homologs of human genes that are likely causal genes in their respective loci according to genome-wide association studies (GWAS) and functional genomic studies: *Apoe*, *Cr1, Bin1, Trem2, Clu, Sorl1, Adam10, Abca7, Cd33, Spi1, Pilra* (Andrews et al. 2020). Volcano plots were made with EnhancedVolcano package (Blighe and Lewis 2018). clusterProfiler package was used for Gene Ontology (GO) and Kyoto Encyclopedia of Genes and Genomics (KEGG) pathway analyses (Kanehisa et al. 2019; Yu et al. 2012).

### Statistical analysis

Shapiro–Wilk normality test was performed, and the results were log-transformed if necessary. One-way ANOVA followed by Tukey’s *post-hoc* test was performed to assess whether the means were different (alpha = 0.05). When we compared the means of two groups, student’s t-test was used, and if the data were non-parametric even after log-transformation, Mann–Whitney test was used. Hedges’ g was calculated with 95% confidence intervals to estimate the effect size of the difference between an anesthetized group and the control group. For comparisons in which the two groups had different standard deviations, Glass’ delta was calculated. All analyses were done on the GraphPad Prism 4.0 software (GraphPad Software, San Diego, CA). Data are expressed as mean ± SEM.

## Results

### Anesthesia induces hyperphosphorylation that returns to normal after 24 h

**Fig. 1:**
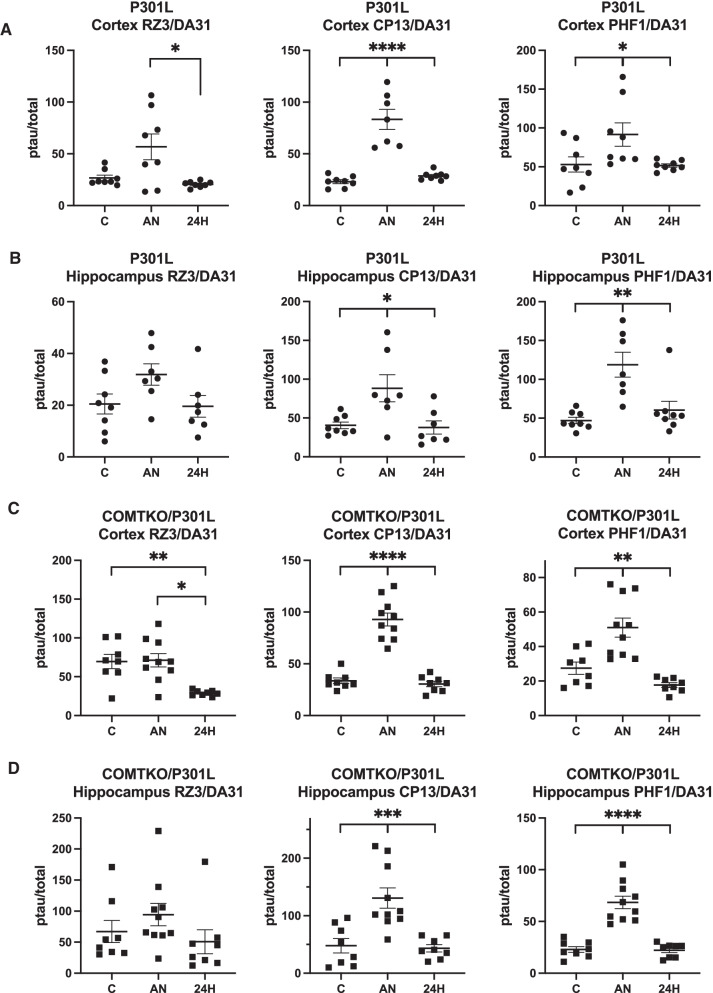
Robust, transient tau hyperphosphorylation after 2-h exposure to isoflurane without temperature-control. Total (DA31) and phosphorylated tau (ptau) in soluble fractions were quantified with ELISA, and the ptau/total tau ratios were compared. **A**, **B** In male 5-month-old P301L mice (n per group = 8, 8, 8/7), we found hyperphosphorylation at the CP13 (Ser202) and PHF1 (Ser396/404) epitopes in the cortex (**A**) and hippocampus (**B**) immediately after anesthesia. **C**, **D** Likewise, in male 4-month-old COMTKO/P301L mice (n per group = 8, 10, 8), we found hyperphosphorylation at the CP13 and PHF1 epitopes in the cortex (**C**) and hippocampus (**D**) immediately after anesthesia. In both models, hyperphosphorylation returned to the level of controls 24 h after anesthesia. Data are mean ± SEM. One-way ANOVA with Tukey’s post-hoc test was performed. *p < 0.05, **p < 0.01, ***p < 0.001, ****p < 0.0001. C, Controls; AN, Anesthesia; 24H, 24-h post-anesthesia

In both mouse models of tauopathy, while total tau was unaltered (Additional file 1: Fig. S1), robust tau hyperphosphorylation followed 2-h administration of isoflurane without temperature control (Fig. 1). Specifically, we found hyperphosphorylation at the CP13 and PHF1 epitopes of soluble tau from both the cortex and hippocampus of 5-month-old P301L mice (Fig. 1A, B) and those of 4-month-old COMTKO/P301L mice (Fig. 1C, D). Effect sizes were modestly greater in the COMTKO/P301L mice immediately after anesthesia (Table 1). But in each model, the hyperphosphorylation in the anesthetized mice returned to the level of controls after 24 h, and the effect sizes significantly decreased (Table 1). These findings suggest that the impact of anesthesia in soluble tau fractions was transient.

**Table 1:** Effect sizes of anesthesia-induced tau hyperphosphorylation immediately and 24 h after anesthesia

	Soluble cortex			Soluble hippocampus		
	RZ3	CP13	PHF1	RZ3	CP13	PHF1
	Effect size (95% CI)			Effect size (95% CI)		
*Immediately after AN*						
COMTKO/P301L (n per group = 8, 10)	0.060 (− 0.87,0.99)	3.57* (2.08,5.07)	1.52* (0.46,2.57)	0.48 (− 0.46,1.42)	1.65* (0.57,2.72)	2.85* (1.53,4.16)
P301L (n per group = 8, 8/7)	1.12* (0.06,2.17)	3.17* (1.65,4.69)	1.01 (− 0.03,2.06)	0.98 (− 0.09,2.06)	1.38* (0.25,2.51)	2.26* (0.97,3.56)
*24 h after AN*						
COMTKO/P301L (n per group = 8, 8)	− 2.07* (− 3.28,− 0.86)	− 0.38 (− 1.37,0.61)	− 1.20* (− 2.27,− 0.14)	− 0.30 (− 1.28,0.69)	− 0.15 (− 1.13,0.83)	− 0.10 (− 1.08,0.89)
P301L (n per group = 8, 8/7)	− 1.00 (− 2.04,0.04)	1.11* (0.05,2.16)	− 0.07 (− 1.05,0.92)	− 0.08 (− 1.09,0.94)	− 0.15 (− 1.16,0.87)	0.52 (− 0.48,1.52)

The level of hyperphosphorylation, as quantified by ELISA, in the mice either immediately or 24 h after anesthesia was compared to that of the control mice. Hedges’ g was calculated with 95% confidence intervals to estimate the effect size of the difference between two groups. Asterisk indicates that the 95% confidence interval does not include zero. Sample size for the control group is listed first in the first column. *AN* anesthesia, *CI* confidence interval

### Anesthesia is associated with transcriptomic changes in genes related to AD pathophysiology

**Fig. 2:**
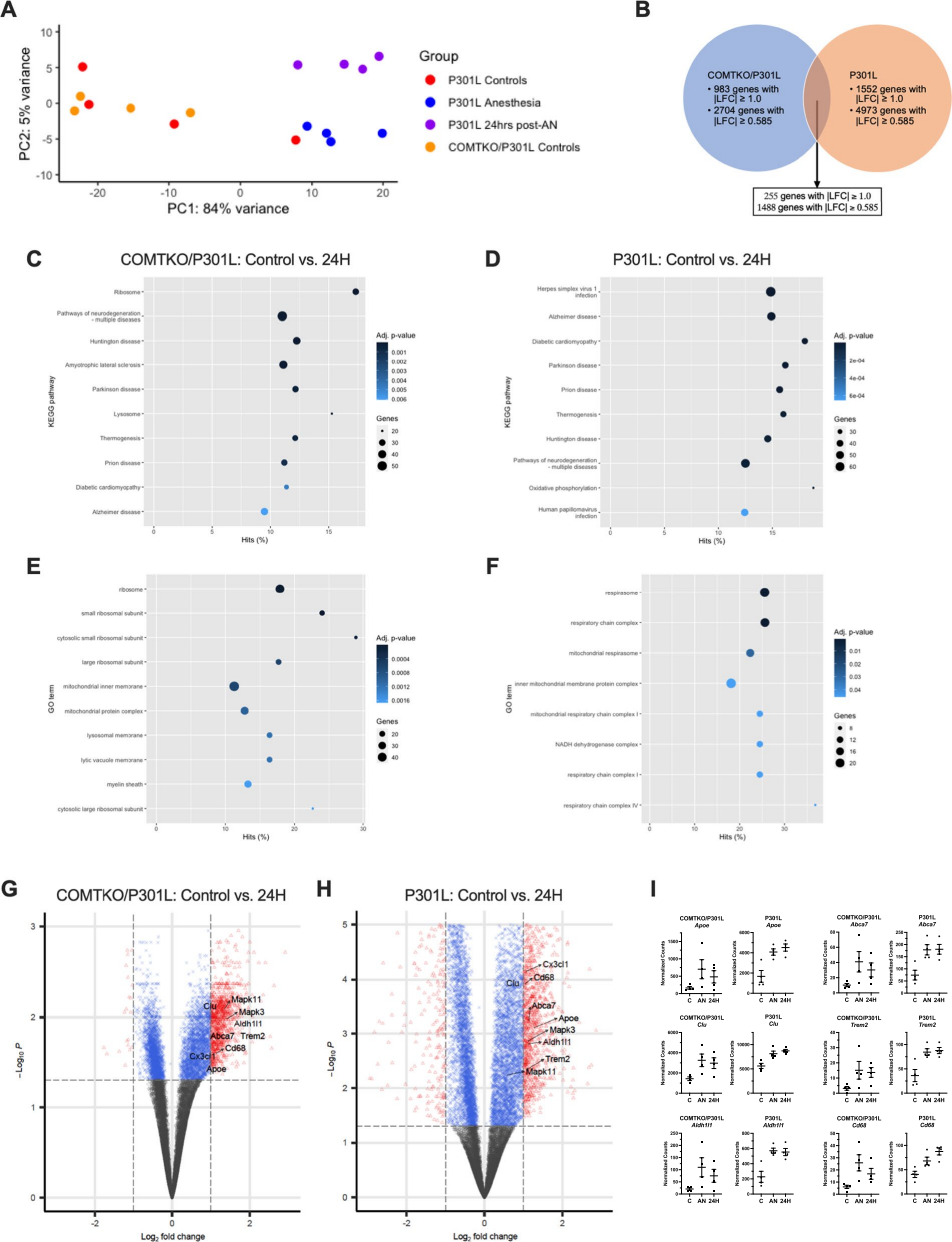
Transcriptomic response in the forebrain of COMTKO/P301L and P301L mice 24 h after isoflurane administration. **A** Principal component analysis showing segregation of anesthetized P301L mice from the control groups of COMTKO/P301L and P301L mice. **B** Venn diagram depicting the number of genes that exceed the absolute log_2_-fold-change (LFC) threshold of 1.0 and 0.585 in the COMTKO/P301L, P301L, and both mouse models. **C**–**F** Kyoto Encyclopedia of Genes and Genomics (KEGG) pathways (**C**, **D**) and Gene Ontology (GO)-cellular component terms (**E**, **F**) that were over-represented in the COMTKO/P301L (**C**, **E**) and P301L (**D**, **F**) mice 24 h after anesthesia. **G**, **H** Volcano plots showing differentially expressed genes in the COMTKO/P301L (**G**) and P301L (**H**) mice 24 h after anesthesia. The x- and y-thresholds indicate the FDR-adjusted p-value of 0.05 and LFC of 1.0, respectively. **I** Comparison of normalized counts of genes that are associated with late-onset AD and are expressed in astrocytes and microglia. The genes were differentially expressed immediately and 24 h after anesthesia. In each mouse model, n = 4 per group. Data are mean ± SEM. *q < 0.05. C, Controls; AN, Anesthesia; 24H, 24-h post-anesthesia

Principal component analysis (PCA) suggested that anesthesia accounted for the largest possible variance among the P301L mice and control COMTKO/P301L mice as the control groups of both mouse models overlapped with one another (Fig. 2A). Based on the PCA analysis, we focused our analyses on gene expression changes that were persistent 24 h after anesthesia when tau hyperphosphorylation in the anesthetized mice returned to the level of controls. Comparison of differentially expressed genes (DEGs) between the COMTKO/P301L and P301L mice revealed 255 genes that were differentially expressed in both models 24 h after anesthesia (Fig. 2B). We defined DEGs as genes that meet the criteria of absolute LFC (|LFC|) greater than or equal to 1.0 (equivalent to fold-change of 2.0 or 0.5) and FDR-adjusted p-value (q-value) lower than 0.05.

Previous studies focused on the activity of protein kinases and phosphatases to elucidate the mechanism of anesthesia-induced tau hyperphosphorylation. In these studies, anesthesia was associated with inhibition of Ser/Thr protein phosphatase 2A (PP2A), an enzyme believed to be involved in tau dephosphorylation (Planel et al. 2007; Whittington et al. 2011). We did not evaluate the activity of PP2A as previous studies did by monitoring tau dephosphorylation (Planel et al. 2007). But PP2A consists of structural A, regulatory B, and catalytic C subunits, and protein assays cannot explore changes in the expression of various subunit isoforms in an unbiased manner (Nematullah et al. 2018). We found overexpression of *Ppp2ca* that encodes the catalytic subunit alpha isoform in the COMTKO/P301L and P301L mice, but it did not reach the fold-change threshold in either model (Additional file 1: Table S1). Instead, we found that overexpression of genes encoding isoforms of structural subunit A (*Ppp2r1a*) and regulatory subunit B (*Ppp2r5b*, *Ppp2r5d*) exceeded at least the lower |LFC| threshold of 0.585 (equivalent to fold-change of 1.5 or 0.66) in both models. In addition, although the activity of tau kinases is generally dependent on phosphorylation states of the proteins for regulation (Cross et al. 1995), we looked at expression of the kinase genes themselves. Among genes encoding canonical kinases that phosphorylate tau (Planel et al. 2007; Reynolds et al. 1997), *Mapk3* and *Mapk11—*which encode ERK1 and p38-β, respectively—had |LFC| greater than 0.585 in both models, providing evidence that they are upregulated in response to anesthesia (Additional file 1: Table S1).

In order to assess the effects of anesthesia on genes and pathways with relevance to delirium and AD, we performed functional over-representation analyses. Transcriptomic data was assessed in an unbiased approach by utilizing computational methodologies that creates gene clusters from experimental data on cellular functions and molecular interactions (KEGG) or from predefined biologic classification systems (GO) (Kanehisa et al. 2019; Yu et al. 2012) (Fig. 2C–F). In both the COMTKO/P301L and P301L mice, KEGG analyses revealed that the top ten over-represented pathways 24 h after anesthesia included those involved in multiple neurodegenerative disease pathogenesis (Huntington’s disease, Parkinson’s disease, prion disease, and Alzheimer’s disease) as well as the thermogenesis pathway that may be directly related to the effects of anesthesia on tau hyperphosphorylation (Fig. 2C, D). GO analyses identified over-representation of cellular component genes related to mitochondrial inner membrane 24 h after anesthesia (Fig. 2E, F). Specifically, mitochondrial genes encoding the electron transport chain (ETC) proteins were differentially expressed in both P301L-based models. The connection between thermogenesis and mitochondria is important as deep anesthesia might be related to reductions in brain metabolism and neurotransmission via inhibition of neuronal mitochondrial activity (Zimin et al. 2018).

We next examined the impact of anesthesia on genes that are thought to be associated with late-onset AD in the COMTKO/P301L and P301L mice. We did not find differential expression of pro- or anti-inflammatory cytokines. Still, at 24-h post-anesthesia, *Apoe* and the gene encoding apolipoprotein E receptor (*Lrp1*) as well as *Trem2, Clu,* and *Abca7* were overexpressed in both models (Fig. 2G–I, Additional file 1: Table S2). Given the role of glial homeostasis in AD (Vogels et al. 2019), we examined genes encoding commonly used markers of astrocytes and microglia, and we found overexpression of *Cd68* and *Aldh1l1* in both mice (Fig. 2G–I, Additional file 1: Table S2). CD68 is expressed across different microglial activation stages in human brains (Hendrickx et al. 2017), while *Aldh1l1* is a marker of astrocytes expressed throughout both white and grey matters (Cahoy et al. 2008). Finally, while the microglial receptor *Cx3cr1* was not differentially expressed, *Cx3cl1*, which is mainly expressed in neurons, was upregulated in both mice (Fig. 2G, H, Additional file 1: Table S2). Chemokine fractalkine (CX3CL1) is the natural ligand of CX3CR1 and involved in modulating microglial activity (Vogels et al. 2019). These data suggest that anesthesia induces the overexpression of genes related to AD pathophysiology in the P301L-based models up to 24 h after anesthesia.

### Hyperphosphorylated insoluble tau increases in the anesthetized mice one and three months after anesthesia

**Fig. 3:**
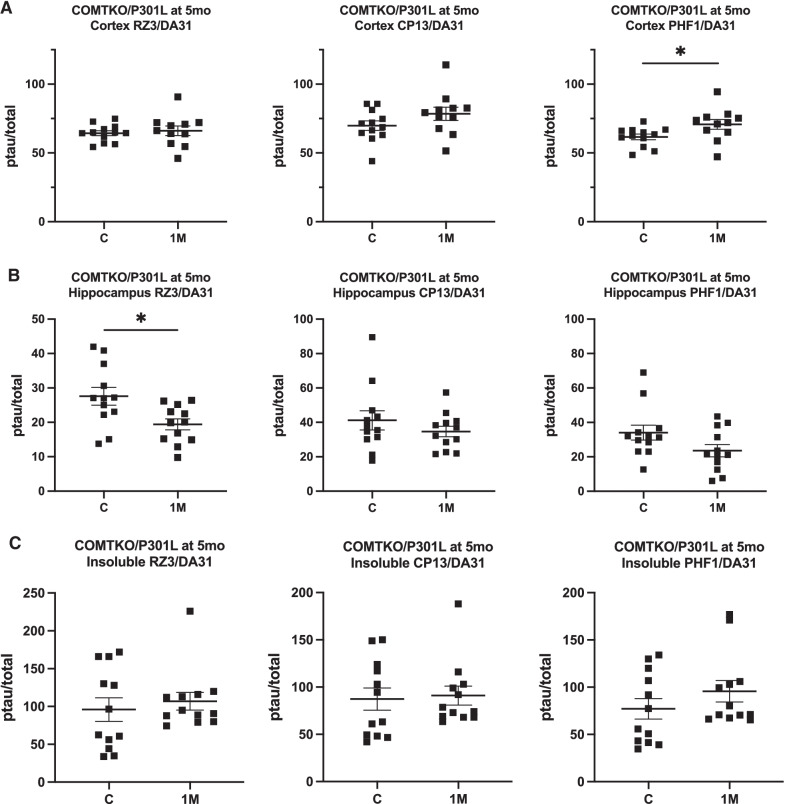
Hyperphosphorylation of soluble and insoluble tau one month after anesthesia in COMTKO/P301L mice. Total (DA31) and phosphorylated tau (ptau) were quantified with ELISA, and the ptau/total tau ratios were compared. **A**, **B** In the soluble fraction of mice that were anesthetized when 4-month-old, phosphorylation at the PHF1 epitope was increased in the cortex (**A**), while that of RZ3 was decreased in the hippocampus (**B**). **C** In the insoluble fraction of the cortex, no differences were found. n per group = 12. Data are mean ± SEM. Student’s t-test was performed. *p < 0.05, **p < 0.01. C, Controls; 1 M, one-month post-anesthesia; mo, month-old

As P301L-based models accumulate tau pathology over their lifespan, we evaluated the latent effects of anesthesia-induced tau hyperphosphorylation on tau biochemistry in the COMTKO/P301L mice at multiple time points, starting at one-month post-anesthesia (Fig. 3, Additional file 1: Fig. S2). In the setting of greater initial anesthesia-induced tau hyperphosphorylation in the COMTKO/P301L mice (Table 1), we were interested in the long-term implications of anesthesia under a hyperdopaminergic state. In the COMTKO/P301L mice anesthetized when 4-month-old, phosphorylation of soluble tau at the PHF1 epitope was increased in the cortex (t(21) = 2.255, p = 0.0350; Fig. 3A), but phosphorylation at the RZ3 epitope was decreased in the hippocampus (t(22) = 2.521, p = 0.0195; Fig. 3B). Insoluble tau from the cortex was utilized as a proxy for aggregated tau, as hyperphosphorylated insoluble tau has similar biochemical properties to the NFTs found in human AD, including resistance to dephosphorylation (Chai et al. 2011; Santacruz et al. 2005). We did not find hyperphosphorylation of insoluble tau or difference in total insoluble tau one month after anesthesia (Fig. 3C, Additional file 1: Fig. S2, Table 2).

**Table 2:** Effect sizes of anesthesia-induced tau hyperphosphorylation one and three months after anesthesia

	Insoluble cortex		
	RZ3	CP13	PHF1
	Effect size (95% CI)		
*One month after AN*			
COMTKO/P301L at 5 mo (n per group = 12)	0.22 (− 0.58, 1.02)	0.09 (− 0.71, 0.89)	0.46 (− 0.35, 1.27)
*Three months after AN*			
COMTKO/P301L at 7 mo (n per group = 17, 13)	2.07* (1.18, 2.96)	2.03* (1.14, 2.91)	1.69* (0.85, 2.53)

The level of hyperphosphorylation, as quantified by ELISA, in the mice either one or three months after anesthesia was compared to that of the control mice. Hedges’ g was calculated with 95% confidence intervals to estimate the effect size of the difference between two groups. Asterisk indicates that the 95% confidence interval does not include zero. Sample size for the control group is listed first in the first column. *AN* anesthesia, *CI* confidence interval, *mo* months old

**Fig. 4:**
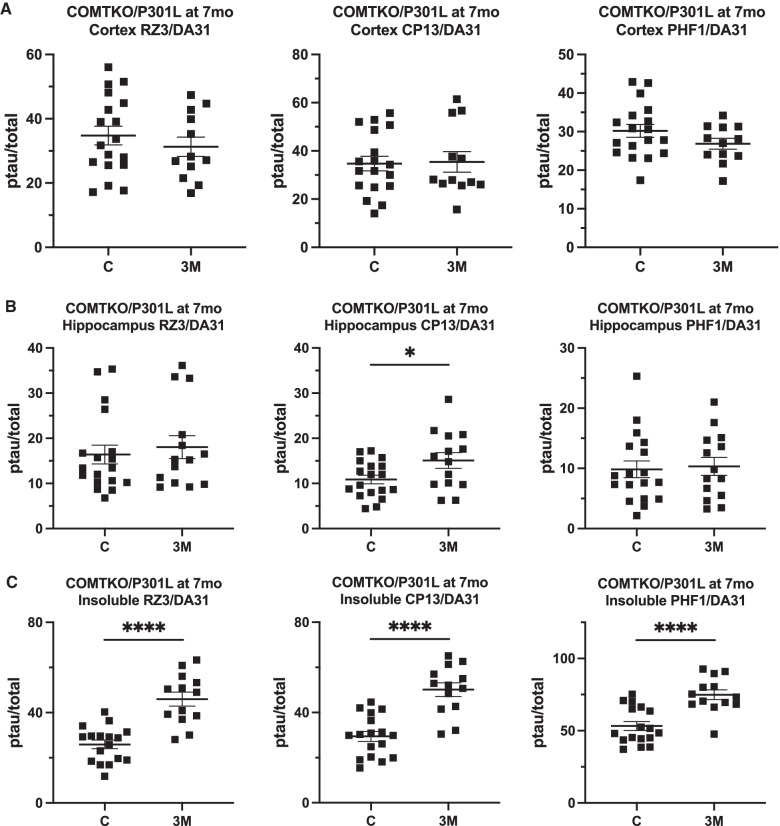
Hyperphosphorylation of soluble and insoluble tau 3 months after anesthesia in COMTKO/P301L mice. Total (DA31) and phosphorylated tau (ptau) were quantified with ELISA, and the ptau/total tau ratios were compared. **A**, **B** In the soluble fraction of mice that were anesthetized when 4-month-old, no differences were found in the cortex (**A**), but phosphorylation at the CP13 epitope was increased in the hippocampus (**B**). **C** In the insoluble fraction of the cortex, phosphorylation was increased at all three phospho-epitopes. n per group = 17, 13. Data are mean ± SEM. Student’s t-test was performed. *p < 0.05, ****p < 0.0001. *C* Controls, *3 M* 3-months post-anesthesia, *mo* month-old

Despite mixed findings at one-month post-anesthesia, we examined whether single anesthetic exposure could alter the long-term trajectory of AD pathology at three-months post-anesthesia. One mouse in the anesthesia group died in between anesthesia and euthanasia. In the anesthetized COMTKO/P301L mice, we did not find hyperphosphorylation of soluble tau in the cortex (Fig. 4A), but phosphorylation of soluble tau at the CP13 epitope was increased in the hippocampus (t(30) = 2.263, p = 0.0310; Fig. 4B). In contrast to the findings at one month, phosphorylation of insoluble tau from the cortex was increased in the anesthetized mice at all three epitopes with effect sizes ranging from 1.69 to 2.07 (Fig. 4C, Table 2): RZ3 (t(28) = 5.779, p < 0.0001), CP13 (t(28) = 5.651, p < 0.0001), and PHF1 (t(28) = 4.713, p < 0.0001). Total insoluble tau was also increased in the anesthetized mice (t(28) = 2.631, p = 0.0137; Additional file 1: Fig. S3). Whereas the younger mice at one-month post-anesthesia showed higher insoluble ptau/total with RZ3, which is the marker of early-stage pathology (Fig. 3C), the older mice at three-months post-anesthesia showed higher insoluble ptau/total with PHF1, which is a late-stage marker (Fig. 4C). These data indicate that the latent effects of single anesthetic exposure on tau pathology might not be apparent until later in the mice’s lifespan when they begin to exhibit motor and behavioral dysfunction (Lewis et al. 2000).

## Discussion

In mouse models of tauopathy, single isoflurane exposure without temperature-control promoted acute tau hyperphosphorylation across brain regions and aggregation of tau at one- and three-months post-anesthesia. While this is the first study to report the latent effects of single anesthetic exposure on tau as far out as three months, previous studies have investigated the extent of anesthesia’s impact on acute tau hyperphosphorylation and tau aggregation after a shorter interval. Early studies in non-transgenic mice suggested that tau hyperphosphorylation following anesthesia may be a consequence of hypothermia, driven by PP2A rather than activation of canonical tau kinases, such as GSK-3β, ERK/MAPK, and p38 (Planel et al. 2007). But in the P301L mice, isoflurane increased insoluble tau aggregates one week after the last exposure, even though neither PP2A inhibition nor kinase activation was associated with the changes after one week (Planel et al. 2009). This suggested that acute hyperphosphorylation has lingering effects on insoluble tau independent of kinase and phosphatase activity. Subsequent studies have demonstrated that tau hyperphosphorylation following anesthesia is independent of hypothermia. For example, propofol hyperphosphorylated tau in non-transgenic mice under both normothermia and hypothermia, albeit to a lesser degree with temperature-control (Whittington et al. 2011). In a triple-transgenic mouse expressing the P301L mutation, repeated isoflurane exposures under normothermic condition were associated with increased phospho-tau immunostaining two months after last exposure (Tang et al. 2011a). Based on the accumulated evidence and our findings from mRNA-sequencing, we hypothesize that the protracted effects of anesthesia-induced hyperphosphorylation might involve both hypothermia-independent and hypothermia-dependent mechanisms.

The transcriptomic responses to anesthesia in the P301L-based models show that anesthesia activates genes that are related to neuroinflammation and implicated in progression of dementia. The mechanism by which apolipoprotein E increases AD risk is unclear (Long and Holtzman 2019), but given its constant presence in GWAS studies, our observation that anesthesia increases the level of *Apoe,* which is expressed by astrocytes, as well as *Lrp1* poses another possible link between anesthesia and AD. The overexpression of genes that are associated with phagocytosis (*Trem2, Abca7*) or are markers of glial responsiveness (*Aldh1l1, Cd68*) raises the question of whether glial response to anesthesia is neuroprotective or neurotoxic (Andrews et al. 2020; Hendrickx et al. 2017; Cahoy et al. 2008; Lambert et al. 2013). In another mouse model with mutant human tau, TREM2 expression increases with age (Jiang et al. 2015), but overexpression might decrease tau hyperphosphorylation and neurodegeneration only at earlier stages of tau pathology (Vogels et al. 2019). Our findings were consistent with previous studies that showed anesthesia alone may not alter the production of pro- or anti-inflammatory cytokines (Schreuder et al. 2017), but we found that *Cx3cl1,* the gene encoding chemokine fractalkine, was upregulated in both P301L-based models immediately and 24 h after anesthesia. Neurons constitutively express high levels of CX3CL1 as it acts on the microglial receptor CX3CR1 to enhance recruitment of homeostatic microglia and inhibit neurotoxic microglia (Vogels et al. 2019; Cardona et al. 2006). Stereotaxic delivery of P301L tau in non-transgenic mice increased neuronal expression of CX3CL1, which subsequently attenuated microglial activation (Lastres-Becker et al. 2014). Extracellular tau competes with soluble CX3CL1 for binding to CX3CR1, and microglial phagocytosis of tau might be dependent on CX3CR1 (Bolós et al. 2017). Meanwhile, in vitro studies have shown that cells secrete hyperphosphorylated tau more efficiently than non-phosphorylated tau (Brunello et al. 2020; Plouffe et al. 2012; Pooler et al. 2012), and elevated tau biomarkers in humans following anesthesia and surgery indicate neuronal release of hyperphosphorylated tau (Evered et al. 2018; Tang et al. 2011b). Taken together, anesthesia-induced hyperphosphorylation of tau might increase extracellular tau that is cleared by microglia, and upregulated *Cx3cl1* might represent a neuroprotective response to the glial phagocytosis. In older individuals with pre-existing tau pathology and impaired microglial response, insufficient clearance of hyperphosphorylated tau might accelerate tau aggregation via mechanisms such as prion-like propagation (Mudher et al. 2017). Our study could not investigate the wide-ranging changes in gene expression at the protein level, but future studies should employ a proteomic approach with subsequent characterization of protein–protein interactions.

Given the over-represented thermogenesis pathway and differential expression of ETC genes, one possible explanation for the temperature-dependent effect of anesthesia might be the demonstrated link between metabolic rate depression and tau hyperphosphorylation in hibernating mammals (Stieler et al. 2011a). During the torpor state characterized by hypothermia and suspension of energy-consuming cellular processes, various brain regions in animals such as bears, squirrels, and hamsters show a transient, reversable hyperphosphorylation of tau (quantified with CP13 and PHF-1) that are associated with altered synaptic activity and cognitive impairment reminiscent of delirium (Stieler et al. 2011a; Arendt et al. 2003). Acute depression of neuronal metabolism by deep anesthesia with isoflurane is thought to activate pathways that are involved in thermoregulation and are phylogenetically related to hibernation in other mammals (Stieler et al. 2011a; Berndt et al. 2021). As a tissue-preservation strategy during torpor, it is believed that regulators of signal transduction cascades, including mitogen activated protein kinases (MAPKs), are engaged to alter gene expression (Storey 2003). Specifically in adipocytes of squirrels, phosphorylated levels of extracellular-signal-regulated kinase ERK1/2, JNK1, and p38 fluctuate significantly over the course of torpor and arousal and are believed to be critical components of non-shivering thermogenesis (Rouble et al. 2014). In hibernating hamsters, alterations in the activity of cdk5 and ERK1/2 were observed in association with tau phosphorylation (Stieler et al. 2011b). In the current study, the genes encoding ERK1 (*Mapk3),* JNK1 (*Mapk8),* and p38 (*Mapk11*), all of which phosphorylate tau (Reynolds et al. 1997; Rouble et al. 2014), were differentially expressed in both models either immediately or 24 h following anesthesia. At the same time, two isoforms of PP2A regulatory B subunit that interact with ERK1/2 and cdk5 were overexpressed (Nematullah et al. 2018; Louis et al. 2011). Our study did not assess the activities of PP2A and tau kinases, but future studies should explore the activity of tau kinases in relation to regulation of PP2A assembly. Whereas hyperphosphorylation of tau may be protective in hibernation (Arendt et al. 2003), a condition in which coterminous expression of heat shock proteins prevent protein aggregation (Storey et al. 2011), a similar cascade of cellular events during anesthesia might have unfortunate consequences for latent tau aggregation in those with pre-existing tau pathology.

Despite the longitudinal follow-up of tau aggregation and novel use of mRNA-sequencing in anesthetized mice, our study has several limitations. We did not include a mouse model that could serve as a negative control to the P301L-based models. We could not use wild-type mice because murine tau undergoes post-translational modifications such as phosphorylation but does not aggregate (Oddo et al. 2003). Mice that have neither murine nor human tau and are on the same background strain as our P301L-based models should be included in future studies. Furthermore, we did not evaluate the effects of anesthesia on NFT formation and behavior. While IHC characterizes morphology and spatial distribution of tau pathology, objective semi-quantification of IHC staining in the cortex is challenging. Not only is the morphology of the cortex more variable than the pyramidal layer of the CA1 hippocampus, but also the stereotypic spread of pathological tau throughout the cortex is unpredictable. In the P301L-based models, development of motor impairment is a significant confounder in behavioral assays that assess spatial memory and learning or other phenotypes. As clinical trials of disease-modifying therapies have shown, improved performances on rodent behavioral assays have not translated to the slowing of cognitive decline, while changes in pathology have demonstrated stronger correlations (Long and Holtzman 2019). Finally, although studies using population-based data and biomarkers of AD have shown anesthesia as a possible risk factor of cognitive decline in older populations regardless of the type of surgery (Chen et al. 2014; Kim et al. 2018; Berger et al. 2016; Evered et al. 2018), effects of surgery cannot be delineated from that of anesthesia in humans. Multiple standardized models of surgery in mice exist (e.g., intracranial, abdominal), but a wide range of predisposing and precipitating risk factors—including infections, metabolic abnormalities, and polypharmacy—are associated with post-operative cognitive decline and limit generalizability. Most preclinical studies of anesthesia used a single rodent model and did not explore possible in vivo modulators, such as endogenous neurochemistry. By comparing the effects of anesthesia between the P301L and COMTKO/P301L mice, we demonstrate consistent patterns in tau hyperphosphorylation and gene expression changes that point to the validity of our model for studying the effects of anesthesia alone.

## Conclusions

Our study lays the groundwork for future studies to investigate the consequences of anesthesia-induced tau hyperphosphorylation and latent tau aggregation in mice. Although acute hyperphosphorylation of tau was transient following anesthesia without temperature-control, changes in expression of genes that are relevant for dementia persisted for 24 h after anesthesia, and hyperphosphorylated tau aggregates were elevated 3 months after anesthesia. New preclinical paradigms that better approximate complications following anesthesia in humans would be helpful for identifying new perioperative and long-term prevention and treatment strategies. Longitudinal human studies focused on biomarkers, including tau imaging post-anesthesia, are necessary to determine whether anesthetic exposure affects tau pathology in tandem with cognition and to ascertain its consequences for AD risk and progression.

## Supplementary Information

**Additional file 1: Fig. S1.** Total tau levels immediately after and 24 hours after 2-hour exposure to isoflurane without temperature-control. Total (DA31) tau in soluble fractions were quantified with ELISA and compared. A-B, In male 5-month-old P301L mice (n per group = 8, 8, 8/7) and male 4-month-old COMTKO/P301L mice (n per group = 8, 10, 8), we did not find any difference in total soluble tau immediately after or 24 hours after anesthesia. Data are mean ± SEM. One-way ANOVA with Tukey’s post-hoc test was performed. C, Controls; AN, Anesthesia; 24H, 24-hours post-anesthesia. **Fig. S2.** Total tau levels one month after anesthesia in COMTKO/P301L. Total (DA31) tau in soluble and insoluble fractions were quantified with ELISA and compared. In the soluble fraction of mice that were anesthetized when 4-month-old, we found decreased level of total tau in the hippocampus. But in both the soluble and insoluble fractions of cortex, we did not find any difference. Data are mean ± SEM. One-way ANOVA with Tukey’s post-hoc test was performed. *p<0.05. C, Controls; 1M, one-month post-anesthesia; mo, month-old. **Fig. S3.** Total tau levels three month after anesthesia in COMTKO/P301L. Total (DA31) tau in soluble and insoluble fractions were quantified with ELISA and compared. In the soluble fraction of mice that were anesthetized when 4-month-old, we did not find any difference in total tau in the cortex or hippocampus. But in the insoluble fraction of the cortex, we found increased level of total tau. Data are mean ± SEM. One-way ANOVA with Tukey’s post-hoc test was performed. *p<0.05. C, Controls; 3M, 3-months post-anesthesia; mo, month-old. **Table S1.** Differential expression of tau kinases and phosphatases immediately after and 24 hours after anesthesia. **Table S2.** Differential expression of genes relevant in late-onset AD and neuroinflammation.

## Data Availability

The datasets used and/or analyzed during the current study are available from the corresponding author on reasonable request.

## Abbreviations

AD: Alzheimer’s disease
NFT: Neurofibrillary tangles
Aβ: Amyloid-β
PHF: Paired helical filaments
CNS: Central nervous system
COMT: Catechol-*O*-methyltransferase
ELISA: Enzyme-linked immunosorbent assay
TBS: Tris-buffered saline
IHS: Immunohistochemistry
LFC: Log_2_-fold-change
GWAS: Genome-wide association studies
PCA: Principal component analysis
DEG: Differentially expressed genes
PP2A: Protein phosphatase 2A
KEGG: Kyoto Encyclopedia of Genes and Genomics
GO: Gene ontology
ETC: Electron transport chain
MAPK: Mitogen activated protein kinase
