# Supplementary material for: Anesthesia promotes acute expression of genes related to Alzheimer’s disease and latent tau aggregation in transgenic mouse models of tauopathy
Source: Mol Med. 2022 Jul 20;28:83. doi: 10.1186/s10020-022-00506-4 (PMC9297560; doi:10.1186/s10020-022-00506-4)
Supplement: Supplementary file 1 — Additional file 1: Fig. S1. Total tau levels immediately after and 24 hours after 2-hour exposure to isoflurane without temperature-control. Total (DA31) tau in soluble fractions were quantified with ELISA and compared. A-B, In male 5-month-old P301L mice (n per group = 8, 8, 8/7) and male 4-month-old COMTKO/P301L mice (n per group = 8, 10, 8), we did not find any difference in total soluble tau immediately after or 24 hours after anesthesia. Data are mean ± SEM. One-way ANOVA with Tukey’s post-hoc test was performed. C, Controls; AN, Anesthesia; 24H, 24-hours post-anesthesia. Fig. S2. Total tau levels one month after anesthesia in COMTKO/P301L. Total (DA31) tau in soluble and insoluble fractions were quantified with ELISA and compared. In the soluble fraction of mice that were anesthetized when 4-month-old, we found decreased level of total tau in the hippocampus. But in both the soluble and insoluble fractions of cortex, we did not find any difference. Data are mean ± SEM. One-way ANOVA with Tukey’s post-hoc test was performed. *p<0.05. C, Controls; 1M, one-month post-anesthesia; mo, month-old. Fig. S3. Total tau levels three month after anesthesia in COMTKO/P301L. Total (DA31) tau in soluble and insoluble fractions were quantified with ELISA and compared. In the soluble fraction of mice that were anesthetized when 4-month-old, we did not find any difference in total tau in the cortex or hippocampus. But in the insoluble fraction of the cortex, we found increased level of total tau. Data are mean ± SEM. One-way ANOVA with Tukey’s post-hoc test was performed. *p<0.05. C, Controls; 3M, 3-months post-anesthesia; mo, month-old. Table S1. Differential expression of tau kinases and phosphatases immediately after and 24 hours after anesthesia. Table S2. Differential expression of genes relevant in late-onset AD and neuroinflammation. [file 10020_2022_506_MOESM1_ESM.docx]

**Figure S1. Total tau levels immediately after and 24 hours after 2-hour exposure to isoflurane without temperature-control**. Total (DA31) tau in soluble fractions were quantified with ELISA and compared. **A-B,** In male 5-month-old P301L mice (n per group = 8, 8, 8/7) and male 4-month-old COMTKO/P301L mice (n per group = 8, 10, 8), we did not find any difference in total soluble tau immediately after or 24 hours after anesthesia. Data are mean ± SEM. One-way ANOVA with Tukey’s post-hoc test was performed. C, Controls; AN, Anesthesia; 24H, 24-hours post-anesthesia.

**Figure S2. Total tau levels one month after anesthesia in COMTKO/P301L**. Total (DA31) tau in soluble and insoluble fractions were quantified with ELISA and compared. In the soluble fraction of mice that were anesthetized when 4-month-old, we found decreased level of total tau in the hippocampus. But in both the soluble and insoluble fractions of cortex, we did not find any difference. Data are mean ± SEM. One-way ANOVA with Tukey’s post-hoc test was performed. **p*<0.05. C, Controls; 1M, one-month post-anesthesia; mo, month-old.

**Figure S3. Total tau levels three month after anesthesia in COMTKO/P301L**. Total (DA31) tau in soluble and insoluble fractions were quantified with ELISA and compared. In the soluble fraction of mice that were anesthetized when 4-month-old, we did not find any difference in total tau in the cortex or hippocampus. But in the insoluble fraction of the cortex, we found increased level of total tau. Data are mean ± SEM. One-way ANOVA with Tukey’s post-hoc test was performed. **p*<0.05. C, Controls; 3M, 3-months post-anesthesia; mo, month-old.

| **Table S1.** **Differential expression of tau kinases and phosphatases immediately after and 24 hours after anesthesia.** | | | | | |  |
| --- | --- | --- | --- | --- | --- | --- |
| Gene Symbol | Gene Name | Control vs. AN  (fold-change) | | Control vs. 24-hours  post-AN  (fold-change) | |  |
|  |  | COMTKO/  P301L | P301L | COMTKO/  P301L | P301L | |
| *Ppp2ca* | PP2A catalytic subunit alpha | 0.811* | 0.736* | 0.916 | 0.739* | |
| *Ppp2r1a* | PP2A 65kDa regulatory subunit alpha | 2.309* | 1.707* | 1.817* | 1.869* | |
| *Ppp2r5b* | PP2A 56kDa regulatory subunit beta | 4.882* | 1.978* | 3.053* | 1.870* | |
| *Ppp2r5d* | PP2A 56kDa regulatory subunit delta | 2.391* | 1.622* | 2.072* | 1.561* | |
| *Akt1* | RAC-alpha ser/thr-protein kinase (AKT1) | 1.280 | 1.702* | 1.094 | 1.785* | |
| *Camk2a/2b* | Calcium/calmodulin-dependent protein kinase type II α chain/β chain (CAMK2A/2B) | 1.213*/  1.512* | 1.473*/  1.628* | 1.088/  1.264 | 1.457*/  1.649* | |
| *Cdk5* | Cyclin dependent kinase 5 (CDK5) | 2.668* | 1.488* | 1.999* | 1.276 | |
| *Gsk3b* | Glycogen synthase kinase 3-β (GSK3B) | 0.756* | 0.810 | 0.760* | 0.847 | |
| *Mapk1* | Extracellular signal-regulated kinase 2 (ERK2) | 0.883 | 0.857 | 0.847* | 0.833* | |
| *Mapk3* | Extracellular signal-regulated kinase 1 (ERK1) | 4.325* | 2.103* | 2.618* | 2.141* | |
| *Mapk8* | Mitogen-activated protein kinase 8 (JNK1) | 0.681* | 0.675* | 0.749* | 0.698* | |
| *Mapk11* | p38-β | 3.033* | 1.836* | 2.546* | 1.472* | |
| *Mapk14* | p38-α | 1.013 | 1.105 | 1.009 | 1.194* | |
| *Prkaa1/2* | AMP-activated protein kinase catalytic subunit α 1/2 | 0.623*  0.687* | 0.672*  0.691* | 0.741*  0.716* | 0.703*/  0.759* | |

Fold-change in expression of genes encoding serine/threonine-protein phosphatase 2A (PP2A) subunits and kinases that phosphorylate tau. We corrected for multiple comparisons using the Benjamini, Krieger, and Yekutieli method for False Discovery Rate (FDR) approach. *, FDR-adjusted p-value < 0.05; AN, anesthesia.

| **Table S2. Differential expression of genes relevant in late-onset AD and neuroinflammation.** | | | | | |
| --- | --- | --- | --- | --- | --- |
| Gene Symbol | Gene Name | Control vs. AN  (fold-change) | | Control vs. 24-hours post-AN  (fold-change) | |
|  |  | COMTKO/P301L | P301L | COMTKO/P301L | P301L |
| *Apoe* | Apolipoprotein E | 3.593* | 2.150* | 1.691* | 2.380* |
| *Trem2* | Triggering receptor expressed on myeloid cells 2 | 2.614* | 2.025* | 3.079* | 2.079* |
| *Clu* | Clusterin | 1.955* | 1.402* | 2.263* | 1.510* |
| *Abca7* | ATP-binding cassette sub-family A member 7 | 3.134* | 2.189* | 2.433* | 2.202* |
| *Aldh1l1* | Cytosolic 10-formyltetrahydrofolate dehydrogenase | 4.362* | 2.247* | 2.871* | 2.143* |
| *Gfap* | Glial fibrillary acidic protein | 1.174* | 1.567* | 1.131 | 1.634* |
| *Aif1* | Allograft inflammatory factor 1 | 2.179* | 1.195 | 1.753* | 1.449* |
| *Cd68* | Cluster of Differentiation 68 | 3.381* | 1.547* | 2.173* | 2.031* |
| *Cx3cl1* | Fractalkine precursor | 1.869* | 1.620* | 1.580* | 2.067* |
| *Cx3cr1* | CX3C chemokine receptor 1 | 1.285* | 1.094 | 1.208 | 1.122 |

Fold-changes are shown for: causal genes of late-onset Alzheimer’s disease; genes encoding commonly used markers of astrocytes and microglia; and genes encoding CX3C chemokine and its receptor. All fold-change values without asterisk are statistically significant after correcting for multiple comparisons using the Benjamini, Krieger, and Yekutieli method for False Discovery Rate (FDR) approach. *, FDR-adjusted p-value < 0.05; AN, anesthesia.
